# Specialties in Medicine

**Published:** 1867-06

**Authors:** 


### SPECIALTIES IN MEDICINE.

The subject indicated by our caption has, for some time, elicited a good share of attention in the "American Medical Association," as well as in other medical societies. In the capacity of reporter for the press, we have visited the meetings of the Cincinnati Academy of Medicine a number of times. The "Academy" is composed exclusively of regular physicians,—graduates in active practice in Cincinnati—at least its active members must be such; and it appears to include in its membership much, and perhaps nearly all the medical talent of the city.

In our visits, though always entertained, generally edified, and often amused, we have not met with much bearing on the interests of Dental surgery directly, and hence our silence in the "Register."

A few weeks ago the subject of specialties was under consideration, and the discussion was led off by Prof. Williams, the occulist and aurist, a professor in the Miami Medical College, and one of the editors of the "Western Lancet and Observer." Of course he advocated specialties, and we are happy to state he did so ably and well. We have heard nothing better on the subject since the lecture of the late Prof. Brainerd, before the Chicago meeting of the "American Dental Association." He was eloquently followed by Prof. Graham, of the Ohio Medical College, who also advocated the practice of specialties; and he was followed by

Prof. Murphy, of the Miami College, and an editor of the "Lancet and Observer," who also approved of the practice of specialties. All three urged a thorough acquaintance with general medicine before resorting to special practice. Specialist empirics received special condemnation from all of them. The subject was laid over for further consideration, Prof. Blackman, of the Ohio Medical College, remarking that he would like to discuss it, being himself a specialist.

Now, we have been thus minute, because this is a subject in which Dental surgeons have a peculiar interest, they being simply practitioners of a specialty. In former years, and the same is true to some extent yet, there was a strong prejudice against special practitioners on the part of the medical profession. The views of the speakers above referred to would indicate that a change is coming, if it has not already arrived. That this prejudice ever obtained a foot-hold is strange. If a man is so modest that he thinks the whole range of medical practice too large a field for his mental capacity, or that he can be more useful in a narrower sphere, is he to be cast out of the society of the faithful? The history of the progress of Dental science affords the strongest argument extant in favor of specialties in medical science. But a few years ago aching teeth were indiscriminately extracted, without mercy, with barbarous, unwieldy instruments worthy of the Spanish Inquisition; and scarce a thought of arresting the ravages of Dental caries ever entered the minds of medical men. Within the recollection of most of us, artificial teeth were resorted to only to improve the appearance and to aid the speech. The state of affairs now is as well known to the reader as to us. Till the practice of Dental surgery became a specialty, the medical profession had failed even to approximate a correct knowledge of the pathology of Dental decay, the most common ailment of afflicted humanity, and one of the most painful. Nor is it sufficient excuse for this want of knowledge that the disease is not a serious one, or that the teeth are unimportant organs. Few agencies do more to deteriorate the race than the diseases of dentition, to say nothing of the sufferings they cause. Defects of dentition are transmitted to offspring from generation to generation, involving defects in the whole process of nutrition. That the profession, the cause of science, and the world, gain much by the adoption of specialties in medicine is illustrated by the connection of the Dental profession with the history of anæsthesia. Let an active mind be restricted to special practice, having time to study the minute details of science, direct and collateral, that bear on his specialty, he is not long content with what is already known on the subject, but launches out for new discoveries. The Dentist having to inflict pain oftener than any other practitioner, it is not strange that he has been more industrious in a search for something to prevent the pain of operations. Having the time, the freedom from the anxieties and great responsibilities of general practitioners, added to the greater frequency of their painful operations, it is not strange that the Dental profession, and not the medical, discovered and developed anæsthesia. Nor is it strange, on the same principle, that when the first successful anæsthetic was abandoned, notwithstanding its great superiority over others for short and severe operations, after a lapse of nearly a quarter of a century, it is re-introduced, and its use revived, with manifold improvements, not by the medical, but by the Dental profession. For the same reasons, the several methods of producing local anæthesia, are the discoveries of Dentists.

We do not know what was said or done at the late meeting of the "American Medical Association" on the subject of specialties. Unfortunately for ourselves, if not for our readers, prior and more pressing engagements prevented our attendance. But from all the indications coming under our observation, it is evident that the idea of specialties is rapidly gaining in favor. At the last meeting of the "Mississippi Valley Association of Dental Surgeons," the subject of specialties in dental surgery occupied a good deal of attention, and was earnestly advocated by several of the leading members of the society. They felt that great good had been done by specializing Dental surgery, and were fully convinced that further good was to be gained by additional subdivisions and specializations.

The success of opthalmic surgery, as a specialty, is an argument second only to that of Dental surgery, in favor of the views here advocated. This was the point mainly dwelt on by Prof.

Williams at the meeting referred to, only he placed the success of the oculists as the paramount argument in this respect, and he may be right. That one argument would be sufficient, if there was no prejudice to be overcome.

All the speakers at the meeting spoken of, dwelt on the great importance of a man being educated thoroughly as a general practitioner, before assuming to practice any specialty. In the main this is the correct doctrine. But if a man has made up his mind to practice Dental, or ophthalmic surgery, it is not essential that he should be as minutely educated in obstetrics as if he intended to go into general practice; yet without a fair knowledge of the anatomy, physiology, and even pathology of the human female, he is not fit for the proper practice of any specialty.

Our Dental colleges all recognize this principle; and their courses on anatomy, physiology, pathology and chemistry, are quite as extended and minute as they ordinarily are in medical schools; and their course on therapeutics is as extensive, in proportion to the number of diseased conditions likely to come under their care. Of the truth of these statements, no one will have doubts, who reads the report of the examinations for degrees, in the March number of the REGISTER. The examinations in the other colleges, for anything we know, are equally rigid and thorough. W.

